# HN1L/AP-2γ/PLK1 signaling drives tumor progression and chemotherapy resistance in esophageal squamous cell carcinoma

**DOI:** 10.1038/s41419-022-05478-1

**Published:** 2022-12-07

**Authors:** Ting-Ting Zeng, Tian-Hao Deng, Zhen Liu, Jia-Rong Zhan, Yuan-Zhen Ma, Yuan-Yuan Yan, Xiao Sun, Ying-Hui Zhu, Yan Li, Xin-Yuan Guan, Lei Li

**Affiliations:** 1 State Key Laboratory of Oncology in South China, Sun Yat-sen University Cancer Center, 510060 Guangzhou, China; 2 The Affiliated Hospital of Hunan Academy of Traditional Chinese Medicine, 410006 Changsha, China; 3 Hunan Academy of Traditional Chinese Medicine, 410006 Changsha, China; 4 Guangdong Provincial Key Laboratory of Malignant Tumor Epigenetics and Gene Regulation, Guangdong-Hong Kong Joint Laboratory for RNA Medicine, Medical Research Center, Sun Yat-sen Memorial Hospital, Sun Yat-sen University, 510120 Guangzhou, China; 5 Nanhai Translational Innovation Center of Precision Immunology, Sun Yat-sen Memorial Hospital, 528200 Foshan, China; 6 Department of Clinical Oncology, Shenzhen Key Laboratory for Metastasis and Personalized Therapy, The University of Hong Kong-Shenzhen Hospital, 518053 Shenzhen, China; 7 Department of Clinical Oncology, The University of Hong Kong, Hong Kong, China

**Keywords:** Oncogenes, Oesophageal cancer

## Abstract

Hematological and neurological expressed 1 like (HN1L) is a newly identified oncogene in lung cancer and hepatocellular carcinoma recently identified by our team, but its roles in the development and treatment of esophageal squamous cell carcinoma (ESCC) remain incompletely cataloged. Here, using ESCC tissue array and public database analysis, we demonstrated that HN1L was highly expressed in ESCC tissues, which was associated with tumor tissue invasion, poor clinical stage and short survival for ESCC patients. Loss- and gain-of-function studies in ESCC cells revealed that HN1L enhances ESCC cell metastasis and proliferation in vitro and in mice models. Moreover, high level of HN1L reduces the sensibility of ESCC cells to chemotherapeutic drugs, such as Docetaxel. Mechanism studies revealed that HN1L activated the transcription of polo-like kinase 1 (PLK1) by interacting with transcription factor AP-2γ, which increased the expression of malignancy related proteins Cyclin D1 and Slug in ESCC cells. Blocking PLK1 with inhibitor BI-2356 abrogated the oncogenic function of HN1L and significantly suppressed ESCC progression by combining with chemotherapy. Therefore, this study demonstrates the vital pro-tumor role of HN1L/AP-2γ/PLK1 signaling axis in ESCC, offering a potential therapeutic strategy for ESCC patients with high HN1L by blocking PLK1.

## Introduction

Esophageal carcinoma (ESCA), including esophageal adenocarcinoma (ADC) and esophageal squamous cell carcinoma (ESCC), is the eighth most common malignancy and the sixth leading cause of death from cancer worldwide [1, 2]. ESCC is the main pathological type of ESCA. Due to the unclear malignant progression mechanism of ESCC, there is still a lack of targeted drugs for ESCC patients, resulting in a 10% five-year survival rate [3]. Chemotherapy is commonly used in the treatment of ESCC, but drug resistance is the main factor for the failure of ESCC treatment. Therefore, there is an urgent need to elucidate the mechanisms underlying ESCC progression and drug resistance to improve prognosis of the patients with ESCC.

Hematological and neurological expressed 1 like (*HN1L*), also known as *JPT2*, or *C16orf34*, is a multifunctional oncogene that we and other groups have recently identified in lung cancer [4], hepatocellular carcinoma [5], and breast cancer [6]. *HN1L* encodes a 190-aa protein and is specifically expressed in human liver, kidney, and generative organs. However, the physiological function of *HN1L* has not been revealed. Recently, high expression of *HN1L* has been proved to promote cancer development. Our research group is the first in the world to explore the oncogenic function of *HN1L* [4, 5]. Overexpression of *HN1L* in lung cancer was significantly associated with larger tumor size and worse survival for patients. Knockdown of *HN1L* inhibited cell proliferation by interfering with MAPK pathway [4]. Next, we also showed that increased HN1L promoted cell proliferation and metastasis by transcriptionally activating AP-2γ/METTL13/TCF3-ZEB1 signaling axis in hepatocellular carcinoma [5]. These results we report are consistent with those of other research groups. Jiao et al. demonstrated that HN1L promoted migration and invasion of breast cancer by increasing the expression of HMGB1 [6]. In addition, HN1L was also in involved in cancer stemness regulation. Liu et al. showed that HN1L enhanced the stemness of triple-negative breast cancer cells by activating LEPR-STAT3 pathway [7]. Moreover, HN1L could also promote stemness by regulating FOXP2/TGF-beta signaling pathway in prostate cancer [8]. These studies suggest the key roles of HN1L in cancer progression by regulating cell growth, metastasis and stemness. However, the role of HN1L in ESCC has not been studied yet. Recently, Wang et al. revealed that HN1L promoted invasion and metastasis of the esophagogastric junction adenocarcinoma [9]. Therefore, these evidences suggest the critical role of HN1L in ESCC.

In the present study, we demonstrated that high HN1L enhanced ESCC cell metastasis and proliferation by activating the transcription of polo like kinase 1 (PLK1) through interaction with transcription factor AP-2γ. Moreover, silence of *HN1L* increased the sensibility of ESCC cells to chemotherapeutic drug Docetaxel and Cisplatin. Inhibition of PLK1 abolished the oncogenic function of HN1L and enhanced the antitumor effect of chemotherapy, which suggests a potential therapeutic strategy for ESCC patients with high HN1L by combining PLK1 inhibitors and chemotherapy.

## Materials and methods

### Clinical samples and cell lines

Primary ESCC and normal esophageal tissue were collected from Linzhou Cancer Hospital (Linzhou, China). All clinical samples used in this study were approved by the Committees for Ethical Review at the Sun Yat-sen University Cancer Center (Guangzhou, China). Written informed consents were obtained from all recruited patients before clinical samples were collected. Human ESCC cell lines KYSE30, KYSE140, KYSE150, KYSE180, KYSE410, and KYSE510 were purchased from the DSMZ (Braunschweig, Germany). Human embryonic kidney cell 293FT was purchased from the ATCC (Manassas, VA). All cells were cultured in high-glucose Dulbecco’s Modified Eagle Medium (DMEM) supplemented with 10% fetal calf serum (FBS, Gibco, Grand Island, NY) at 37 °C with 5% CO_2_.

### Immunohistochemistry (IHC) staining

ESCC tissue array was made by our research group, and IHC staining was performed as described previously [5, 10]. Firstly, paraffin-embedded tissue slices were placed in an oven at 65 °C and baked for 2 h. For deparaffinization, slices were soaked in pure xylene for three times (15 min per time). Next, the tissue slices were rehydrated with a concentration gradient of alcohol (100, 95, 75, and 50%, 5 min per time) and in the deionized water for 3 min. For antigen retrieval, the hydrated tissue sections were immersed in boiling EDTA Antigen Retrieval Solution (pH 8.0, #P0085, Beyotime, Shanghai, China) for 30 min. The slides were cooled naturally to room temperature and washed with phosphate buffer saline (PBS) for three times (5 min per time). To avoid nonspecific binding, the slides was treated with 5% bull serum albumin (BSA, Amresco, Boise, ID) at 37 °C for 30 min. Primary antibodies against HN1L (1:2000 dilution, #HPA041908, Sigma, Burlington, MA), Ki67 (1:400 dilution, #ab16667, Abcam, Cambridge, MA) and Cleaved Caspase-3 (1:200 dilution, #9661, Cell Signaling Technology, Danvers, MA) were incubated at 4 °C overnight in a humidified chamber. On the second day, the humidified chamber was placed at room temperature for 10 min, and then the slides were washed with PBS for three times (5 min per time). HRP-conjugated secondary antibody (#K5007, Dako, Copenhagen, Denmark) was incubated at 37 °C for 30 min, and the slides were detected with DAB substrate system (#K346711-2, Dako, Santa Clara, CA). Cell nuclei were counterstained with hematoxylin (#TA-125-MH, Thermo Fisher Scientific, Waltham, MA), and the results of IHC staining was observed and imaged under a light microscope (Olympus, Lake Success, NY).

### Immunofluorescent (IF) staining

ESCC cells on coverslips were fixed with 4% paraformaldehyde (#P0099, Beyotime, Shanghai, China). For permeabilization, cells were treated with 0.1% Triton X-100 (#ST795, Beyotime, Shanghai, China), and then the slides were washed with PBS for three times (5 min per time). Cell slides were blocked with 5% BSA at 37 °C for 30 min to avoid nonspecific binding. Primary antibodies against HN1L (1:2000 dilution, #HPA041908, Sigma, Burlington, MA), pan-Cytokeratin antibody (1:100 dilution, #ab86734, Abcam, Cambridge, MA), PLK1 (1:200 dilution, #ab17057, Abcam, Cambridge, MA) and Ki67 (1:400 dilution, #ab16667, Abcam, Cambridge, MA) were incubated at 4 °C for 12 h in a moist chamber. Next, the slides were washed with PBS for three times (5 min per time) and then were incubated with Donkey anti-Rabbit IgG (H + L) Highly Cross-Adsorbed Secondary Antibody, Alexa Fluor™ 594 (1:400 dilution, #A-21207, Thermo Fisher Scientific, Waltham, MA) or Donkey anti-Mouse IgG (H + L) ReadyProbes™ Secondary Antibody, Alexa Fluor™ 488 (1:400 dilution, #R37114, Thermo Fisher Scientific, Waltham, MA). Finally, cell nuclei were mounted with Mounting Medium with DAPI (#ab104139, Abcam, Cambridge, MA). The staining results were imaged with a Laser Scanning Confocal Microscopy (Olympus, Lake Success, NY).

### Lentivirus-mediated HN1L overexpression and knockdown

Lentiviral expression vector pLenti6 (Invitrogen, Carlsbad, CA) containing the coding sequence of human *HN1L* and lentiviral interference vector psi-LVRU6GP (GeneCopoeia, Rockville, MD) with short hairpin RNA (shRNA) targeting *HN1L*, *AP-2γ* or *PLK1* (Table S1) were co-transfected with three lentivirus packaging vectors, including pLp1, pLp2 and pLp-VSVG (Invitrogen, Carlsbad, CA) into 293FT cells (Invitrogen, Carlsbad, CA) with HilyMax transfection reagent (#H357, Dojindo, Japan), respectively. Empty vector pLenti6 or psi-LVRU6P containing scrambled shRNA was performed the same operation as controls. After the virus packaging was completed, the ESCC cells were infected with lentivirus. Stable cell lines were selected by Puromycin (Sigma, Burlington, MA) treatment for two weeks. Western blot was used to confirm HN1L expression at the protein level.

### Protein extraction and western blotting

The culture medium was poured out and cells were washed twice with pre-cooled PBS. Cell proteins were extracted using cold 1× RIPA buffer (#9806, Cell Signaling Technology, Danvers, MA) supplemented with protease inhibitor cocktail (#4693159001, Roche, Basel, Switzerland) and phosphatase inhibitor PhosSTOP (#490683700, Roche, Basel, Switzerland). The protein concentration was measured with BCA Protein Assay Kit (#KGPBCA, KeyGEN, Nanjing, China). Western blotting was performed as described previously [11]. The primary and second antibodies were listed in Table S2. The protein expression levels were analyzed with Chemiluminescence Imaging System (Bio-Rad, Hercules, CA).

### In vitro cell growth assay

ESCC cells with *HN1L* overexpression or knockdown were cultured in 100 μL normal medium and seeded into 96-well plates (2000 cells per well). After 6 h of cell seeding, cell proliferation was detected using the Cell Counting Kit-8 (#CK04, Dojindo, Japan) according to the user instructions. In brief, 10 μL CCK-8 solution was added to each well, and the cells were further incubated in cell culture chambers for 2 h. Absorbance was measured at 450 nm by absorbance photometer (BioTek Instruments, Winooski, VT).

### BrdU incorporation assay

BrdU Cell Proliferation Assay Kit (#11299964001, Roche, Basel, Switzerland) was used to explore cell growth regulated by *HN1L* overexpression or *PLK1* silence. In detail, 1× BrdU solution was added to culture medium, and ESCC cells were further incubated in cell culture chambers for 30 min. BrdU positive cells were detected by IF staining with primary antibody against BrdU and Alexa Fluor™ 594 secondary antibody (1:400 dilution, #A-21203, Thermo Fisher Scientific, Waltham, MA). The cell nuclei were mounted with anti-fade reagent with DAPI (#ab104139, Abcam, Cambridge, MA) before imaging by fluorescence microscope (Olympus, Lake Success, NY).

### Transwell migration assay

ESCC cells in logarithmic growth phase were added to 0.1% FBS culture medium and starved for 24 h. The cells were digested by trypsin and centrifuged at 1000 rpm for 5 min. The cell pellet was suspended with serum-free culture medium, and cell number was counted. A total of 1 × 10^5^ cells were diluted into 0.5 mL serum-free culture medium and added into the upper compartment of the Transwell. Normal culture medium with 10% FBS (0.5 mL) was added into the lower compartment of the Transwell. Cells were cultured at 37 °C and 5% CO_2_ for 48 h. Invasive cells were fixed with alcohol for 10 min and stained with crystal violet for 30 min. Invasive cells were photographed and counted under a light microscope (Olympus, Lake Success, NY).

### Protein co-immunoprecipitation (Co-IP)

ESCC cells were washed three times with cold PBS and lysed with cell lysis buffer NP-40 (#P0013F, Beyotime, Shanghai, China) at ice for 30 min. The protein solution was obtained by centrifugation (14,000 rpm, 4 °C, 15 min). Protein Co-IP was performed using the Dynabeads™ Protein A Immunoprecipitation Kit (#10006D, Invitrogen, Carlsbad, CA) and primary antibody against HN1L (#ab200571, Abcam, Cambridge, MA). The protein levels of HN1L and AP-2γ with Co-IP were analyzed with western blotting.

### Luciferase reporter assay

Luciferase reporter assay was performed as described previously [12]. The putative *PLK1* promoter (NCBI ID: 5347) that contains the binding sites for the transcription factor AP-2γ was inserted upstream of Gaussia Luciferase coding sequences in the pEZX-PG04 basic reporter plasmid (GeneCopoeia, MD, USA). Human AP-2γ coding sequences were cloned into the pEZ-M02 plasmid (GeneCopoeia, MD, USA). KYSE30 cells were co-transfected with pEZ-AP-2γ and pEZX-promoter reporter plasmids using HilyMax transfection reagent (#H357, Dojindo, Japan). KYSE30 cells were seeded in 96-well plates (2 × 10^3^ cells per well) 48 h after transfection. Gaussia luciferase activity was detected with the Secrete-Pair™ Gaussia Luciferase Assay Kit (#LF061, Promega, Madison, WI) according to the technical manual.

### Subcutaneous xenograft tumor assay

The animal study was approved by Animal Ethics Committee at Sun Yat-sen University Cancer Center (Guangzhou China). Four-week-old male BALB/C nude mice were purchased from the Guangdong Medical Laboratory Animal Center (Guangzhou China) and were randomly assigned to experimental groups. ESCC cells (2 × 10^6^/mouse) in 100 μL pure DMEM were subcutaneously transplanted to the right side of the mouse with sterile syringes. Four weeks after injection, the mice were sacrificed with euthanasia. The weights of xenograft tumors were measured with electronic scales.

### Lung metastasis in mice

The cells at the logarithmic growth stage were digested by trypsin, and centrifuged at 1000 rpm for 5 min. A total of 1 × 10^6^ cells were injected into 4-week-old female BALB/C nude mice through the tail vein. The volume of cell suspension was 100 μL for each nude mouse. Two months later, the mice were sacrificed for cervical dislocation, and both lungs were removed to observe the number of nodules on the lung surface. The lung tissues with metastatic tumors were fixed and embedded, then sequentially sectioned, stained with H&E, and observed under a microscope to confirm the presence of tumor cells.

### Statistical analysis

Data analyses were performed using GraphPad Prism 8 (San Diego, CA). The research data between two groups were analyzed with two-sided independent Student’s *t* test. Gene expression levels in ESCA were obtained from the TCGA database using GEPIA 2 (http://gepia2.cancer-pku.cn/) [13]. Gene Ontology analyses of HN1L were performed using Coexpedia (http://www.coexpedia.org/) [14]. *P* value <0.05 was considered statistically significant.

## Result

### Increased HN1L was associated with poor prognosis of ESCC patients

**Fig. 1:**
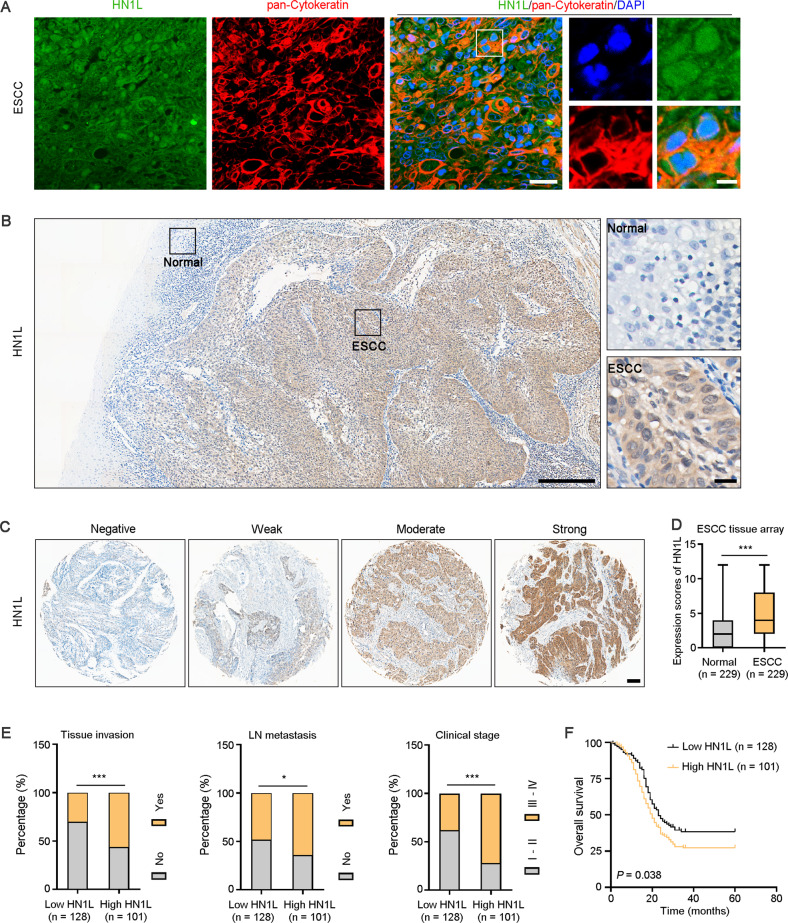
High expression of HN1L predicates poor prognosis in patients with ESCC. **A** Double IF staining showed the cellular localization of HN1L in ESCC tissues. Pan-cytokeratin was used to indicate cancer cells. Scale bar, left 20 μm; right 5 μm. **B** Representative image of IHC staining for HN1L in a clinical sample with normal esophageal epithelial tissue and ESCC tissue. Scale bar in left panel, 300 μm; right panel, 20 μm. **C** IHC staining in the ESCC tissue array showed the different expression levels of HN1L. Scale bar, 100 μm. **D** Expression scores of HN1L in ESCC tissue array (*n* = 229). Data are presented as the mean ± SD; two-sided Student’s *t* test; ***P < 0.001. **E** Correlation analysis between HN1L expression and clinicopathological data. One-way ANOVA; *P < 0.05, ***P < 0.001. **F** Kaplan–Meier survival curves showed that a high level of HN1L was associated with poor prognosis in patients with ESCC.

The Cancer Genome Atlas (TCGA) database analysis revealed that the mRNA level of *HN1L* was higher in ESCC (*n* = 95) than that in ADC (*n* = 89) and normal tissues (*n* = 11) (Fig. S1A). To explore the protein expression and localization of HN1L in ESCC tissues, we performed IF and IHC staining on ESCC sections. Results showed that HN1L was mainly expressed in the nucleus and cytoplasm of ESCC cells (Fig. 1A). And HN1L was highly expressed in ESCC tissues than that in adjacent esophageal epithelial tissues (Fig. 1B). Using the ESCC tissue array (*n* = 229), we displayed that the intensity of HN1L expression varied in different samples (Fig. 1C). The expression score of HN1L was analyzed in terms of staining intensity and the proportion of positive cells. The analysis confirmed the high score of HN1L in ESCC, compared with paired normal esophageal epithelial tissues (*P* < 0.001, Fig. 1D). Correlation analysis of clinicopathological data showed that high level of HN1L was associated with tumor tissue invasion, lymph node metastasis, and poor clinical stage (Fig. 1E). However, there was no correlation between HN1L expression and gender, age, or tumor differentiation (Fig. S1B). Kaplan–Meier analysis showed that ESCC patients with high expression of HN1L had a worse overall survival than those with low level of HN1L (*P* = 0.038, Fig. 1F). These clinical data indicated the oncogenic role of HN1L in ESCC progression.

### High expression of HN1L drives ESCC cell metastasis

**Fig. 2:**
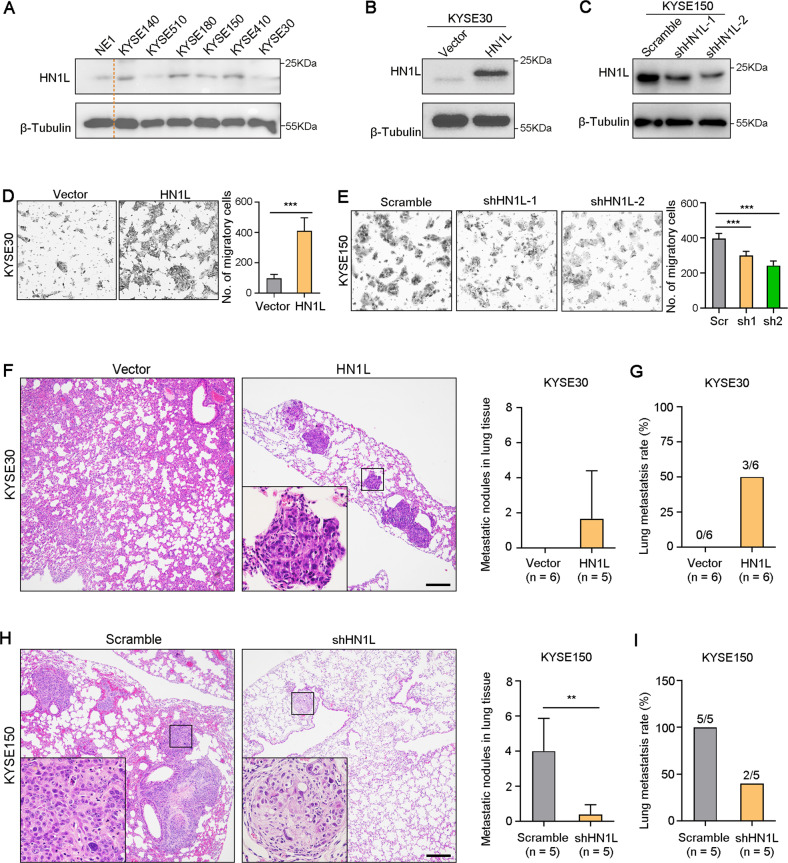
Elevated HN1L enhances ESCC metastasis. **A** HN1L expression in normal esophageal epithelial cell NE1 and ESCC cell lines were analyzed by western blotting. **B** Western blotting was used to confirm the exogenous overexpression of HN1L in KYSE30 cells. **C** Silence of *HN1L* in KYSE150 cell with shRNA was confirmed by western blotting analysis. **D** Transwell assay using KYSE30-Vector and KYSE30-*HN1L* cells. **E** Transwell assay with KYSE150-scramble and KYSE150-sh*HN1L* cells. **F** Lung metastasis of KYSE30-Vector and KYSE30-*HN1L* cells were tested by tail vein injection in nude mice and H&E staining of lung tissues was performed. Scale bar, 250 μm. **G** The rate of mice with lung metastasis was counted. **H** H&E staining of lung tissues from nude mice after tail vein injection of KYSE150-scramble and KYSE150-sh*HN1L* cells. Scale bar, 250 μm. **I** The rate of mice with lung metastasis was counted. In all panels, data are presented as the mean ± SD; two-sided Student’s *t* test; **P < 0.01, ***P < 0.001.

To confirm the pro-tumor role of HN1L in ESCC, we constructed ESCC cell lines with continuous overexpression or knockdown of *HN1L*. Firstly, we analyzed the protein level of HN1L in six ESCC cell lines and one immortalized esophageal epithelial cell line NE1 with western blotting. Results showed that the protein expression of HN1L in KYSE140, KYSE150, KYSE180 and KYSE410 cells was relatively higher than that in NE1 cell (Fig. 2A). KYSE30 and KYSE510 cells with low expression of HN1L were selected for exogenous overexpression of *HN1L* (Fig. 2B). Meanwhile, KYSE150 and KYSE180 cells with a high level of HN1L were transfected with lentivirus-packaged shRNA for *HN1L* knockdown (Fig. 2C). Transwell assay showed that overexpression of *HN1L* enhanced the migration activity of ESCC cells (Figs. 2D and S2A). Inversely, silencing *HN1L* with two shRNAs inhibited ESCC cells migration in vitro (Figs. 2E and S2B). In addition, lung metastasis experiment in nude mice was performed by tail intravenous injection of ESCC cells with overexpression or knockdown of *HN1L*. Hematoxylin and Eosin staining was used to confirm lung metastasis two months after cell injection. Results showed that no lung metastases were found in KYSE30-Vector group, and overexpression of *HN1L* in KYSE30 cells drove the formation of lung metastases (Fig. 2F, G). Significantly, knockdown of *HN1L* in KYSE150 cells decreased the frequency of lung metastasis (Fig. 2H, I). Moreover, ESCC cells with overexpression or knockdown of *HN1L* were injected subcutaneously into the left plantar to evaluate inguinal lymph node metastasis. Results showed that increased *HN1L* in KYSE30 cells drove lymph node metastasis (Fig. S3A–C). Inversely, decreasing the expression of *HN1L* in KYSE150 cells inhibited lymph node metastasis (Fig. S3D–F). Therefore, these results suggested that HN1L has the function of promoting tumor metastasis.

### Overexpression of HN1L promotes ESCC cell proliferation

**Fig. 3:**
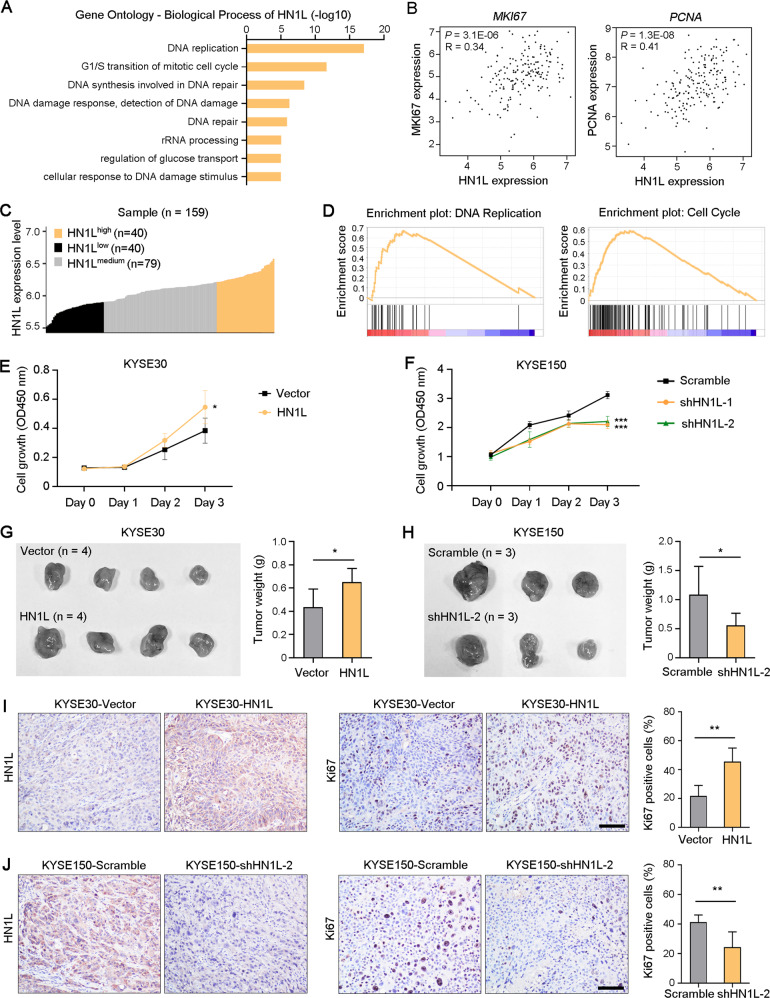
Enhanced HN1L promotes ESCC growth. **A** Gene Oncology analysis (biological process) of *HN1L* with Coexpedia database (https://www.coexpedia.org/). **B** Co-expression analysis between *HN1L* and the markers of cell proliferation (*MKI67* and *PCNA*) in esophageal carcinoma (ESCA) using TCGA cohort. **C** ESCA samples were divided into three groups based on the level of HN1L expression. **D** Pathway enrichment analysis of *HN1L*. **E** Cell growth assay of KYSE30 with vector or *HN1L* transfection. **F** Cell growth assay of KYSE150 with *HN1L* silence. **G** KYSE30-Vector and KYSE30-*HN1L* cells were transplanted subcutaneously into nude mice, and xenograft tumor weights were counted in the right panel. **H** Xenograft tumor experiment was performed using KYSE150-Scramble and KYSE150-sh*HN1L* cells. **I**, **J** IHC staining with antibodies against HN1L and Ki67 was performed respectively on xenograft tumors derived from KYSE30 with *HN1L* overexpression (**I**) or KYSE150 with *HN1L* silence (**J**). Scale bar, 50 μm. In all panels, data are presented as the mean ± SD; two-sided Student’s *t* test; *P < 0.05, **P < 0.01, ***P < 0.001.

Gene Oncology analysis showed that *HN1L* was also involved in DNA replication and repair (Fig. 3A). Correlation analysis of gene mRNA levels using the TCGA database indicated that there were significant positive correlations between *HN1L* level and the markers of cell proliferation *MKI67* and *PCNA* in the ESCA cohort (Fig. 3B). These ESCA samples were divided into three groups based on the level of *HN1L* expression, and pathway enrichment analysis showed that *HN1L* was involved in DNA replication and cell cycle processes (Fig. 3C, D). These bioinformatics analyses suggested that *HN1L* played a key role in cell proliferation. Hence, we analyzed the growth rate of ESCC cells with *HN1L* overexpression or knockdown in vitro. Results showed that overexpression of *HN1L* promoted ESCC cell proliferation (Fig. 3E). Conversely, decreasing the expression of *HN1L* reduced the growth rate of ESCC cells (Fig. 3F). Next, KYSE30 cells with vector or *HN1L* overexpression were transplanted subcutaneously into nude mice, and xenograft tumor weights were analyzed four weeks after cell injection. The heavier weight of KYSE30-*HN1L* group suggested that overexpressed *HN1L* promoted tumor growth (Fig. 3G). A relatively light weight of tumors derived from KYSE150 cells with *HN1L* knockdown was found (Fig. 3H). IHC staining confirmed the high expression of HN1L and proliferation marker Ki67 in KYSE30-*HN1L* cell-derived xenograft tumors (Fig. 3I). Low expressions of both HN1L and Ki67 were indicated in tumors originating from *HN1L*-silenced KYSE150 cells (Fig. 3J), suggesting the oncogenic role of HN1L in ESCC progression by enhancing cell proliferation.

### HN1L enhances chemotherapy resistance of ESCC cells

**Fig. 4:**
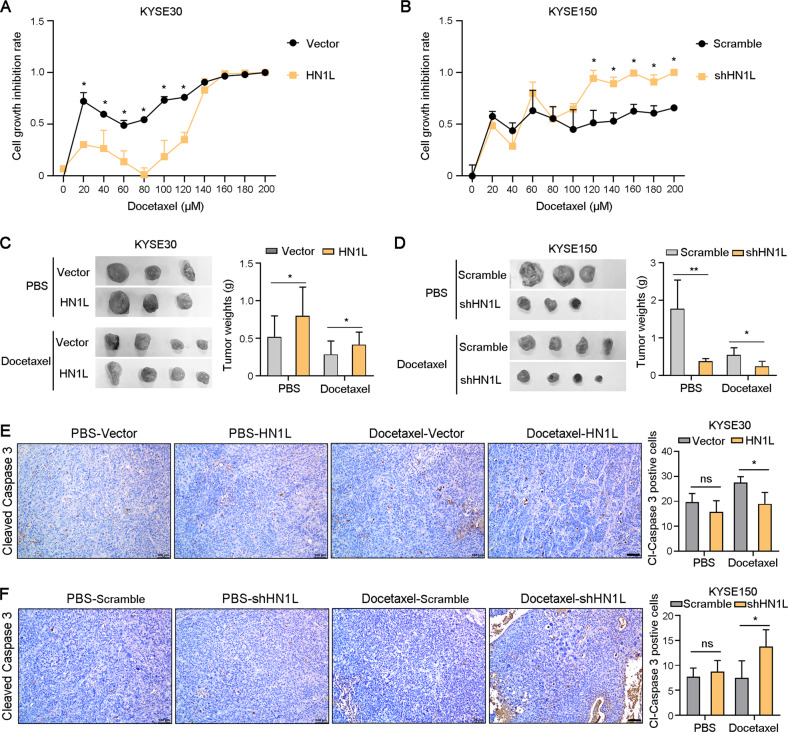
HN1L reduces ESCC cells sensitivity to paclitaxel. **A** The growth inhibition rate of KYSE30-Vector and KYSE30-*HN1L* cells under treatment of Docetaxel. **B** The growth inhibition rate of KYSE150-Scramble and KYSE30-sh*HN1L* cells under treatment of Docetaxel. **C** KYSE30-Vector and KYSE30-*HN1L* cells were transplanted subcutaneously into nude mice and treated with Docetaxel. Xenograft tumor weights were counted in the right panel. **D** Xenograft tumor experiment was performed using KYSE150-scramble and KYSE150-sh*HN1L* cells and treated with Docetaxel. **E**, **F** IHC staining with antibodies against cleaved Caspase 3 was performed on xenograft tumors. And the number of cleaved Caspase 3-positive tumor cells was counted. Scale bar, 100 μm. In all panels, data are presented as the mean ± SD; two-sided Student’s *t* test; *P < 0.05, **P < 0.01, ns: no significant difference.

To explore the role of HN1L in ESCC drug resistance, the growth inhibition rate of KYSE30-Vector and KYSE30-*HN1L* cells under treatment of Docetaxel in vitro was analyzed. Results showed that overexpression of *HN1L* reduced the sensitivity of KYSE30 cell to Docetaxel (Fig. 4A). In contrast, silence of *HN1L* enhanced the inhibitory effect of Docetaxel on cell growth in KYSE150 cell (Fig. 4B). Moreover, KYSE30-Vector and KYSE30-*HN1L* cells were injected subcutaneously into nude mice and Docetaxel treatment was performed 1 week after cell transplantation. Overexpression of *HN1L* promoted xenograft tumor growth and reduced the inhibitory effect of Docetaxel in vivo (Fig. 4C). Inversely, knockdown of *HN1L* inhibited tumor growth and enhanced the sensitivity of KYSE150 cells to Docetaxel (Fig. 4D). The level of apoptotic cells in xenograft tumors was analyzed by IHC staining with antibodies against Cleaved Caspase 3. Overexpression *HN1L* decreased the apoptosis level of ESCC cells under treatment of Docetaxel, and reducing *HN1L* expression increased the level of Docetaxel-induced apoptosis (Fig. 4E, F). In addition, overexpression *HN1L* in KYSE30 cell also reduced the anti-tumor activity of Cisplatin in vivo (Fig. S4A). In contrast, knockdown of *HN1L* improved the sensitivity of tumor cells to Cisplatin (Fig. S4B). Hence, targeting HN1L enhanced the anti-tumor effect of chemotherapeutic drugs.

### PLK1 mediated the oncogenic function of HN1L in ESCC

**Fig. 5:**
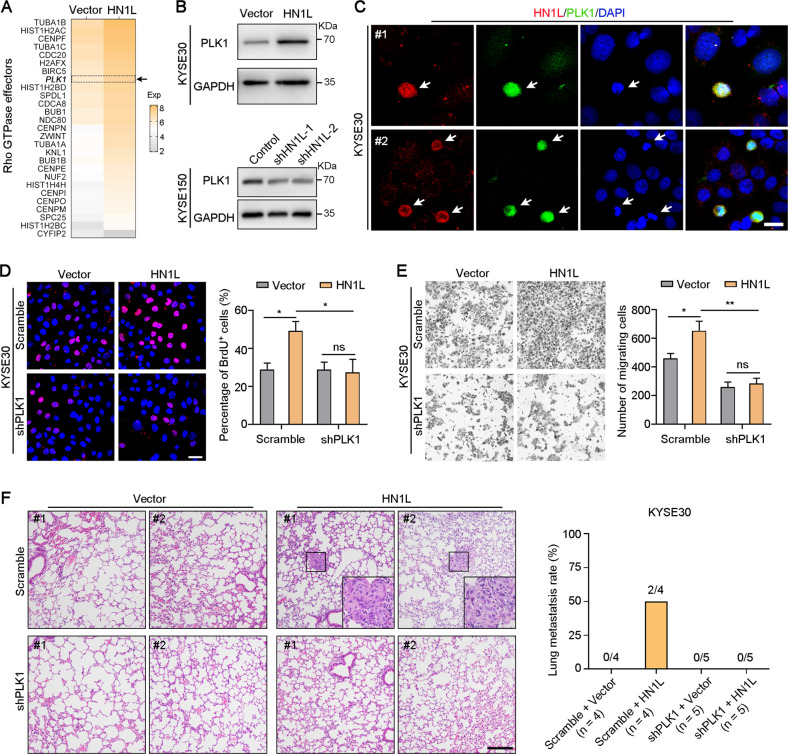
HN1L drives ESCC cell growth and migration by up-regulating PLK1. **A** The heat map showed the expressions of Rho GTPase effectors after *HN1L* overexpression. **B** Western blotting was used to confirm the expression of PLK1 after *HN1L* overexpression or silence. **C** Double IF staining showed the co-expression of HN1L and PLK1 in KYSE30 cells. Scale bar, 20 μm. **D** BrdU incorporation assay showed the cell proliferation rate of KYSE30-Vector and KYSE30-*HN1L* after *PLK1* silence. Scale bar, 20 μm. **E** Transwell migration assay was performed to test the cell migration ability of KYSE30-Vector and KYSE30-*HN1L* after *PLK1* knockdown. **F** Lung metastasis experiment showed that overexpression of HN1L enhanced the metastatic ability of KYSE30 cells, and PLK1 knockdown reduced the metastasis rate of KYSE30-HN1L cells in the lung. Scale bar, 250 μm. In **D**, **E**, data are presented as the mean ± SD; two-sided Student’s *t* test; *P < 0.05, **P < 0.01, ns: no significant difference.

RNA sequencing was performed on KYSE30-Vector and KYSE30-*HN1L* cells to explore the oncogenic mechanisms of *HN1L* in ESCC. Total of 1872 genes were down-regulated and 1296 genes were up-regulated after *HN1L* overexpression (Fig. S5A). KEGG pathway analysis showed that *HN1L* was involved in cell cycle regulation (Fig. S5B). Results of Reactome pathway analysis indicated that *HN1L* was associated with cell cycle checkpoints and Rho GTPase signaling (Fig. S5C). *PLK1* as a member of Rho GTPase effectors was up-regulated after *HN1L* overexpression (Fig. 5A). We focused on PLK1 mainly because of its significant positive regulation of cell proliferation, migration and chemotherapy resistance in ESCC [15, 16]. Moreover, western blotting analysis also confirmed the increased expression of PLK1 after *HN1L* overexpression and PLK1 down-regulation after *HN1L* knockdown at protein level (Fig. 5B). Double IF staining showed the consistent expression of HN1L and PLK1 in proliferative KYSE30 cells in vitro (Fig. 5C). In addition, the mRNA levels of *PLK1* and *HN1L* are positively correlated in ESCA in the TCGA dataset (Fig. S5D). The expression of *PLK1* was higher than that in normal esophageal tissues (Fig. S5E). Gene Ontology and Gene Set enrichment analyses suggested that *PLK1* was involved in DNA replication and cell cycle regulation (Fig. S5F–H). BrdU incorporation assay showed a higher proliferation rate of KYSE30 cells after *HN1L* overexpression, and the pro-proliferation activity of *HN1L* was attenuated after *PLK1* silence (Fig. 5D). Transwell assay indicated that the cell migration ability of KYSE30 cells was enhanced by *HN1L* overexpression, and *PLK1* knockdown reduced the migration ability of KYSE30-*HN1L* cells (Fig. 5E). To further confirm the role of HN1L/PLK1 signaling pathway in ESCC metastasis, we performed the lung metastasis experiment in mice by tail vein injection. Results showed that PLK1 knockdown reduced the metastasis rate of HN1L-overexpressed KYSE30 cells in vivo (Fig. 5F). Rescue experiments showed that PLK1 overexpression rescued the growth rate and migration ability of HN1L-silenced ESCC cells (Fig. S5I, S5J). Therefore, HN1L promoted cancer progression by up-regulating the expression of PLK1.

### HN1L increased the expression of PLK1 by activating AP-2γ

**Fig. 6:**
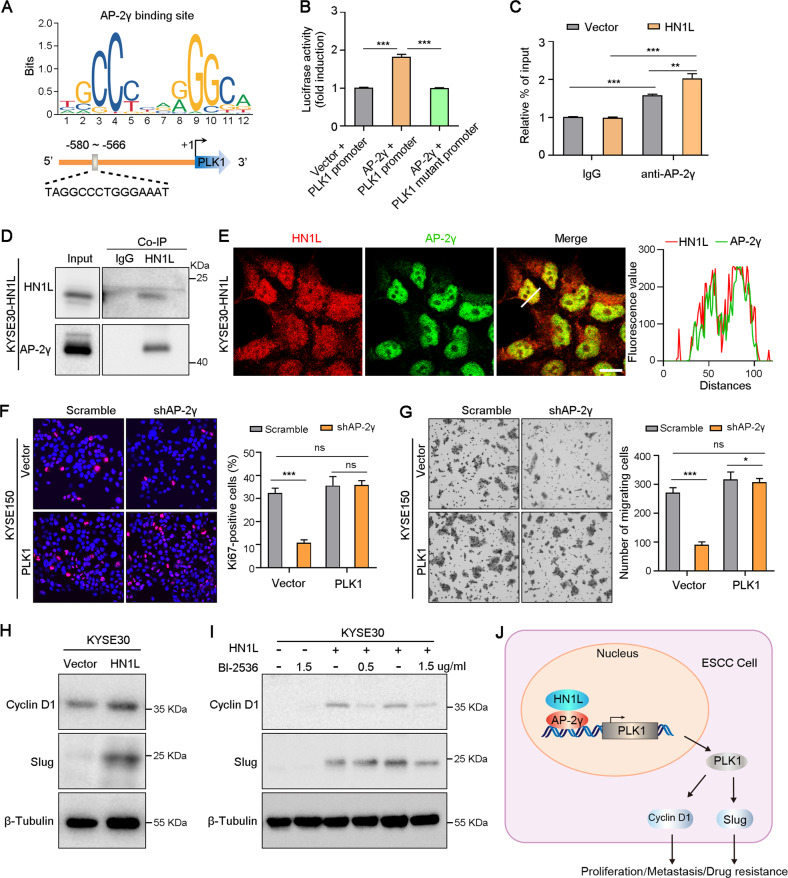
HN1L-AP-2γ-PLK1 axis promotes ESCC progression via up-regulation of Cyclin D1 and Slug. **A** AP-2γ transcriptional binding sites in the *PLK1* gene promoter. **B** Luciferase reporter assay showed that HN1L transcriptionally up-regulated *PLK1* expression by activating AP-2γ in KYSE30 cells. **C** ChIP-qPCR assay to validate that AP-2γ bound to the promoter of PLK1 in Vector or HN1L-transfected KYSE30 cells. **D** Co-IP was performed with HN1L antibody on KYSE30-*HN1L* cells. **E** Double IF staining with antibodies against HN1L and AP-2γ in KYSE30-*HN1L* cells. **F**, **G** The rescue experiments on cell proliferation (**F**) and metastasis (**G**) mediated by PLK1 were performed in AP-2γ-silenced KYSE150 cell. **H** The protein levels of Cyclin D1 and Slug in KYSE30 cells after *HN1L* overexpression were analyzed with western blotting. **I** The protein levels of Cyclin D1 and Slug in KYSE30 cells after *HN1L* overexpression or PLK1 inhibitor BI-2536 treatment were analyzed with western blotting. **J** The diagram shows that HN1L enhances PLK1 expression by activating transcription factor AP-2γ, which further increases Cyclin D1 and Slug expression levels, promoting ESCC cell proliferation, metastasis and drug resistance. In **B**, **C**, **E**, **F**, data are presented as the mean ± SD; two-sided Student’s *t* test; *P < 0.05, **P < 0.01, ***P < 0.001, ns: no significant difference.

We then investigated the molecular mechanism by which HN1L up-regulates PLK1 in ESCC cells. First, we surveyed protein-protein interaction using the IntAct database (https://www.ebi.ac.uk/intact/), and found that HN1L protein potentially interacts with transcription factor AP-2γ (Fig. S6A and Table S3). The mRNA level *TFAP2C* that codes AP-2γ is significantly overexpressed in ESCA tissues (Fig. S6B). We analyzed the transcription factor binding sites in the promoter region of the *PLK1* gene and found a conservative binding sites of AP-2γ upstream of the transcription start site (−580 to −566 nucleotides) (Fig. 6A). Luciferase reporter assay confirmed that *PLK1* expression was transcriptionally up-regulated by AP-2γ in KYSE30 cells (Fig. 6B). ChIP-qPCR assay showed that HN1L promoted AP-2γ binding to the promoter regions of *PLK1* (Fig. 6C). Moreover, Co-IP assay (Fig. 6D) and Double IF staining (Fig. 6E) showed that HN1L bound directly to AP-2γ in KYSE30-*HN1L* cells. QPCR and Western blotting analyses confirmed that AP-2γ knockdown resulted in the down-regulation of PLK1 at mRNA and protein levels (Fig. S6C, D). Rescue experiments showed that PLK1 overexpression rescued the growth rate, migration ability and chemotherapy resistance of AP-2γ-silenced ESCC cells (Figs. 6F, G and S6E). These evidences indicated that HN1L increased *PLK1* expression by activating AP-2γ.

Next, the expressions of cell cycle regulator *CCND1* and metastasis-related transcription factor *SLUG* that have been reported to be the target genes of PLK1 signaling was analyzed using TCGA cohort [17]. The results of correlation analyses showed that the expression levels of *CCND1* or *SLUG* were positively correlated with *HN1L*, *PLK1* or *TFAP2C* in ESCA at the mRNA level (Fig. S6F). Moreover, western blotting also confirmed that knockdown of HN1L, AP-2γ or PLK1 leaded to the decreased expression of Cyclin D1 and Slug in KYSE150 cells (Fig. S6G, H). Overexpression of HN1L increased the protein levels of Cyclin D1 and Slug in KYSE30 cells (Fig. 6H). Inhibition of PLK1 activity with inhibitor BI-2536 can attenuate the up-regulation of HN1L on Cyclin D1 and Slug (Fig. 6I). Cyclin D1 and Slug are known to mediate tumor proliferation, metastasis and drug resistance [18–20]. Therefore, HN1L promoted ESCC progression by enhancing AP-2γ/PLK1/CCND1-SLUG signaling (Fig. 6J).

### Targeting PLK1 weakens the enhancement of HN1L on ESCC drug resistance

**Fig. 7:**
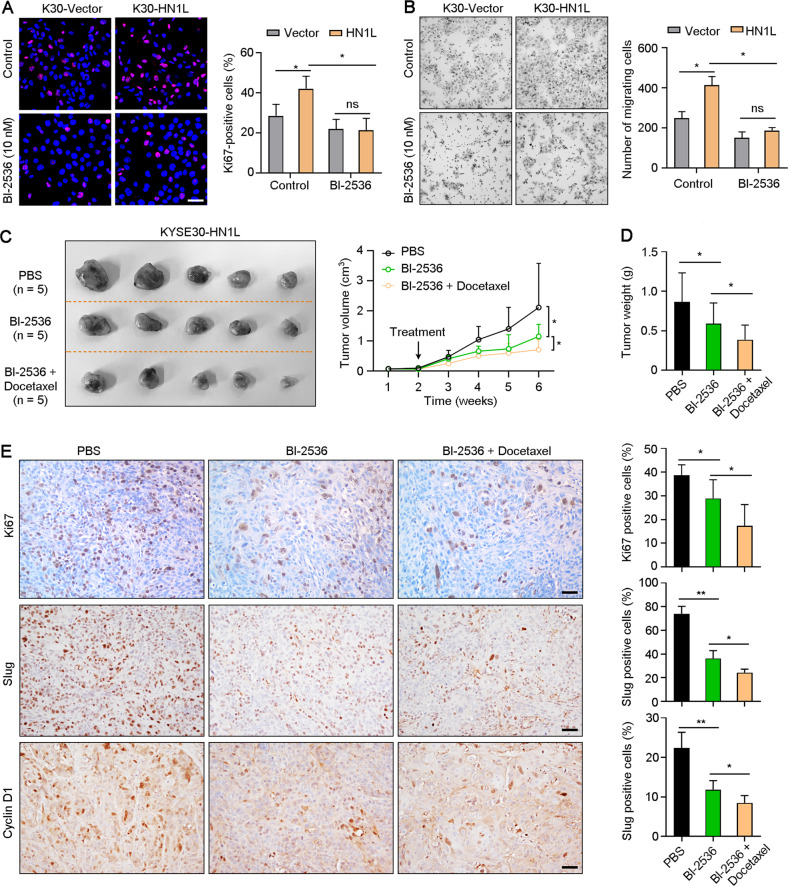
Targeting PLK1 increases the sensitivity of ESCC cells to chemotherapy. **A** IF staining of Ki67 showed the cell proliferation rate of KYSE30-Vector and KYSE30-*HN1L* under treatment of PLK1 inhibitor BI-2536. Scale bar, 20 μm. **B** Transwell migration assay was performed to test the cell migration ability of KYSE30-Vector and KYSE30-*HN1L* under treatment of BI-2536. **C** Xenograft tumors from KYSE30-*HN1L* cells were treated with BI-2536 (5 mg/kg) and Docetaxel (3 mg/kg) 2 weeks after cell injection. Medication was given twice a week for 2 weeks. Tumor volumes (**C**) and weights (**D**) were counted. **E** IHC staining with antibodies against Ki67, Slug, and Cyclin D1 was performed on xenograft tumors. Scale bar, 50 μm. In all panels, data are presented as the mean ± SD; two-sided Student’s *t* test; *P < 0.05, **P < 0.01, ns: no significant difference.

To explore the feasibility of targeting HN1L/PLK1 signaling in the treatment of ESCC, PLK1 inhibitor BI-2536 was used to treat KYSE30 cells with or without *HN1L* overexpression in vitro and in vivo. First, results of IF staining with antibody against Ki67 indicated that inhibition of PLK1 abolished the function of HN1L to promote ESCC cell proliferation (Fig. 7A). Transwell assay was performed to analyze the cell migration ability of KYSE30-Vector and KYSE30-*HN1L* under treatment of BI-2536. Results showed that the migration ability of KYSE30-*HN1L* cells was abolished by PLK1 blocking (Fig. 7B). Next, to explore whether targeting PLK1 can improve the sensitivity of ESCC with high expression of *HN1L* to chemotherapy drugs, KYSE30 cell with *HN1L* overexpression was injected subcutaneously into nude mice and treated with BI-2536 (5 mg/kg) and Docetaxel (3 mg/kg). Tumor volumes and weights analyses showed that BI-2536 treatment inhibited tumor growth and enhanced the anti-tumor effect of Docetaxel (Fig. 7C, D). IHC staining indicated the expression levels of Ki67, Slug and CyclinD1 were decreased after treatment with BI-2536 and Docetaxel, compared with BI-2536 alone or PBS control (Fig. 7E). Therefore, PLK1 inhibitor combined with chemotherapy may be a promising treatment for ESCC patients with a high level of HN1L.

## Discussion

*HN1L* shares 30% identity with *HN1* and belongs to the *HN1* family, which is an evolutionarily conserved gene family [21]. *HN1L* gene encodes a 20-kDa protein, which is localized in both the nucleus and cytoplasm. The normal physiological function of *HN1L* is unknown. However, high expression of *HN1* has been proved to contribute to proliferation, migration and invasion in many malignancies, such as breast cancer [22], prostate cancer [23, 24], thyroid carcinoma [25] and hepatocellular carcinoma [26]. Recently, *HN1L*, as a homolog of *HN1*, has also been gradually confirmed to promote tumor progression by promoting tumor growth, metastasis and stemness in various human cancers [4–9]. These studies suggested that the members of the *HN1* gene family play a vital role in cancer development. Therefore, this study continued to explore the oncogenic function and molecular mechanism of *HN1L* in ESCC, providing new evidence for targeted *HN1L* therapy of tumors. Gain- and loss-of-function studies in ESCC cells revealed that overexpression of *HN1L* could not only promote cell proliferation and metastasis, but also enhance cell chemotherapy resistance. Knockdown of *HN1L* increased the sensibility of ESCC cells to Docetaxel and Cisplatin. This study is the first to investigate the role of *HN1L* in chemotherapy. Revealing the mechanisms underlying the oncogenic role of *HN1L* will provide new therapeutic targets and combination therapies for patients with ESCC.

Current studies have gradually revealed the carcinogenic mechanism of *HN1L*. We firstly demonstrated that knockdown of *HN1L* induced cell cycle arrest by inhibiting the MAPK pathway via interaction with RASA4 protein in lung cancer [4]. In hepatocellular carcinoma, we have revealed that HN1L could act as a transcription activator and activate the transcription factor AP-2γ, which further promoted tumor growth and metastasis [5]. AP-2γ, coding by gene *TFAP2C*, is highly expressed in cancer tissues and was involved in cancer progression [27, 28]. The activation and expression of AP-2γ could be regulated through N6-methyladenosine modification [29], miRNA [30] or LncRNA [31]. Wong et al. showed that AP-2γ-Myc-KDM5B protein complex promoted cell cycle progression via direct p21 repression in breast cancer [32]. Those studies indicated that the transcriptional activity of AP-2γ was regulated by other transcriptional regulatory factors. In this study, we also proved that HN1L was directly bound to AP-2γ using Co-IP assay, which transcriptionally activated the expression of Rho GTPase effector *PLK1* in ESCC cells (Fig. 6D). At present the functional domains of HN1L are completely unknown. Therefore, it is difficult to accurately identify the binding sites of HN1L and AP-2γ. Although the details of how HN1L bind and activate AP-2γ are still unclear, the key role of HN1L in ESCC necessitates further study on its specific mechanisms. Therefore, HN1L promoted ESCC progression by activating AP-2γ/PLK1 signaling, which was a novel cancer-promoting mechanism of HN1L.

Rho GTPases is the largest subfamily of the Ras-homology superfamily. Most Rho GTPases exist in active GTP form or a GDP-bound inactive conformation to regulate their activity to regulate cellular processes, including cell migration, cell polarity and cell proliferation progression [33]. Accumulating evidences exemplify the importance of Rho effectors in multi-steps of cancer progression, and PLK1 is one of them [34]. PLK1 is proverbial to regulate cell proliferation, migration and invasion via phosphorylation of specific substrates [35]. Moreover, inhibition of PLK1 kinase enhances the chemosensitivity of cisplatin by inducing pyroptosis in ESCC [36]. Overexpression of PLK1 has been found in many human cancers, which was associated with poor survival in cancer patients [37]. Therefore, PLK1 was selected as the target gene of HN1L for further study. In the percent study, we showed that PLK1 as a key downstream target of the HN1L/AP-2γ pathway promoted ESCC progression by up-regulation of cell cycle-related gene *CCND1* and metastasis regulatory gene *SLUG*. These results are supported by other studies. Montaudon et al. found that PLK1 inhibition resulted in tumor shrinkage and metastasis inhibition in advanced *CCND1*-driven breast cancer [38]. Slug, encoding by *SLUG* gene, is a well-known transcription factor in promotion of cancer metastasis and drug resistance [39]. Hence, HN1L/AP-2γ/PLK1/CCND1-SLUG signaling axis drives ESCC progression and chemotherapy resistance, suggesting that blocking PLK1 may attenuate the oncogenic role of HN1L.

BI-2536 is a small molecule inhibitor of PLK1 and significantly reduced cell viability by inducing cell cycle arrest and cell apoptosis in neuroblastoma [40, 41] and triple-negative breast cancer [42]. We found that inhibition of PLK1 with BI-2536 or siRNA suppressed ESCC cell proliferation and migration enhanced by *HN1L* overexpression. These findings indicated that PLK1-targeted cancer therapy was more appropriate ESCC in patients with high *HN1L* expression. Moreover, it has been proved that BI-2536 treatment enhanced the chemosensitivity of cisplatin by inducing pyroptosis in ESCC [36]. Combination of PLK1 inhibition with chemotherapeutic drugs also enhanced the sensitivity toward Taxol [43–45]. Our in vivo xenograft tumor experiments have showed that the combination of BI-2536 and Paclitaxel has a better therapeutic effect than the drug alone in *HN1L*-overexpressed ESCC cells (Fig. 7C). We reasoned that inhibition of PLK1 could reverse drug resistance mediated by HN1L and increase the activity of chemotherapeutic drugs.

Taken together, this study reports a HN1L-mediated mechanism used by ESCC cells to promote cell proliferation, metastasis and drug resistance by activating AP-2γ/PLK1 signaling pathway. HN1L services as a poor prognostic factor in ESCC and a significant biomarker for selecting patients who benefit from chemotherapy. PLK1 inhibition in combination with chemotherapy may be a promising therapeutic strategy for ESCC patients with high HN1L.

## Supplementary information

Supplementary Figure Legends

Supplementary Figure S1

Supplementary Figure S2

Supplementary Figure S3

Supplementary Figure S4

Supplementary Figure S5

Supplementary Figure S6

Supplementary Table S1

Supplementary Table S2

Supplementary Table S3

Supplementary materials-RNA sequencing data

Raw data of western blots

Reproducibility checklist

## Data Availability

The published article includes all data analyzed for this study.
