# Supplementary material for: HN1L/AP-2γ/PLK1 signaling drives tumor progression and chemotherapy resistance in esophageal squamous cell carcinoma
Source: Cell Death Dis. 2022 Dec 7;13(12):1026. doi: 10.1038/s41419-022-05478-1 (PMC9729194; doi:10.1038/s41419-022-05478-1)
Supplement: Supplementary file 1 — Supplementary Figure Legends [file 41419_2022_5478_MOESM1_ESM.docx]

**Supplementary Figure Legends**

**Fig. S1 High expression of HN1L in ESCC tissue.** **(A)** The mRNA level of *HN1L* was higher in ESCC than that in esophageal adenocarcinoma (ADC) and normal tissues in TCGA database. **(B)** HN1L expression has no correlation with gender, age or tumor differentiation. ****P* < 0.001, ns: no significant difference.

**Fig. S2 HN1L promotes ESCC cell migration in vitro. (A)** Transwell migration assay was performed on KYSE510-Vector and KYSE5100-*HN1L* cells. **(B)** Transwell migration assay with KYSE180-scramble and KYSE180-sh*HN1L* cells. In all panels, data are presented as the mean ± SD. ****P* < 0.001.

**Fig. S3 High HN1L promotes lymph node metastasis in nude mice.** **(A)** Inguinal lymph node (red arrow) was obtained from nude mice four weeks after subcutaneous injection of KYSE30-Vector or KYSE30-*HN1L* cell into the left plantar. **(B)** Image of lymph nodes. **(C)** Hematoxylin-Eosin staining of lymph nodes. Scar bar, upper 500 μm; lower 100 μm. **(D)** Inguinal lymph node (red arrow) was obtained from nude mice four weeks after subcutaneous injection of KYSE150-Scramble or KYSE150-sh*HN1L* cell into the left plantar. **(E)** Image of lymph nodes. **(F)** Hematoxylin-Eosin staining of lymph nodes. Scar bar, upper 500 μm; lower 100 μm. In panels **B** and **E**, data are presented as the mean ± SD. **P* < 0.05.

**Fig. S4 HN1L reduces the sensitivity of ESCC cells to Cisplatin (DDP).** **(A)** KYSE30-Vector and KYSE30-*HN1L* cells were transplanted subcutaneously into nude mice and treated with DDP (5mg/kg). **(B)** Xenograft tumor experiment was performed using KYSE150-scramble and KYSE150-sh*HN1L* cells and treated with DDP (5mg/kg). Xenograft tumor weights were counted in the right panel. In all panels, data are presented as the mean ± SD. **P* < 0.05.

**Fig. S5 HN1L up-regulates PLK1 in ESCC.** **(A)** RNA sequencing was performed on KYSE30-Vector and KYSE30-*HN1L* cells, and the Volcano plot showed the genes with significant differences. **(B)** KEGG pathway analysis after *HN1L* overexpression. **(C)** Results of Reactome pathway analysis. **(D)** The mRNA levels of *PLK1* and *HN1L* have a positive correlation in ESCA in the TCGA dataset. **(E)** The expression of *PLK1* was higher than that in normal esophageal tissues. **(F)** Gene Ontology enrichment analysis of *PLK1*. **(G)** ESCA samples were divided into three groups based on the level of *PLK1* expression. **(H)** Pathway enrichment analysis of *PLK1* using TCGA database. (**I, J**) The rescue experiments on cell proliferation (**I**) and metastasis (**J**) mediated by PLK1 were performed in HN1L-silenced KYSE150 cell. In panels **E, I** and **J**, data are presented as the mean ± SD. **P* < 0.05, ****P* < 0.001, ns: no significant difference.

**Fig. S6 HN1L up-regulates CCND1 and SLUG in ESCA by interacting AP-2γ.** **(A)** The interaction network of HN1L obtained from IntAct database (https://www.ebi.ac.uk/intact/home). **(B)** The mRNA level of *AP-2γ* in ESCA and normal esophageal epithelial tissues. **P* < 0.05. **(C)** QPCR analysis showed that knockdown of *HN1L* or *AP-2γ* reduced the expression of *PLK1* at mRNA level. **(D)** Western blotting also confirmed that *AP-2γ* knockdown resulted in the down-regulation of PLK1 at protein level. **(E)** chemotherapy resistance experiments in AP-2γ-silenced KYSE150 cell with or without PLK1 overexpression. **(F)** Correlation analysis between *HN1L*, *PLK1*, *TFAP2C* and target genes *CCND1* or *SLUG* in ESCA tissues using TCGA datasheet. **(G)** QPCR analysis was used to confirm the silence of PLK1 in KYSE150 cell. **(H)** Western blotting was performed to analyze the protein levels of Cyclin D1 and Slug in KYSE150 cells after knockdown of *HN1L*, *AP-2γ* or *PLK1*. In panels **B, C, E** and **J**, data are presented as the mean ± SD. **P* < 0.05, ***P* < 0.01, ****P* < 0.001, ns: no significant difference.
