# Supplementary figures and images for: HN1L/AP-2γ/PLK1 signaling drives tumor progression and chemotherapy resistance in esophageal squamous cell carcinoma
Source: Cell Death Dis. 2022 Dec 7;13(12):1026. doi: 10.1038/s41419-022-05478-1 (PMC9729194; doi:10.1038/s41419-022-05478-1)

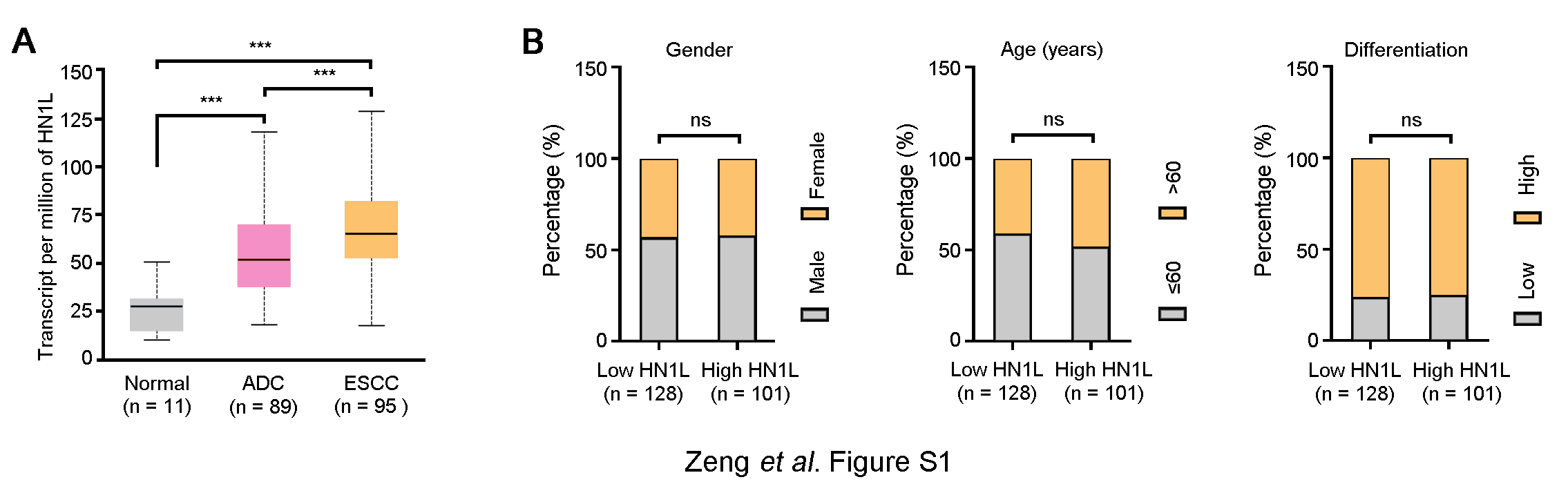

Supplement: Supplementary file 2 — Supplementary Figure S1 [file 41419_2022_5478_MOESM2_ESM.tif]

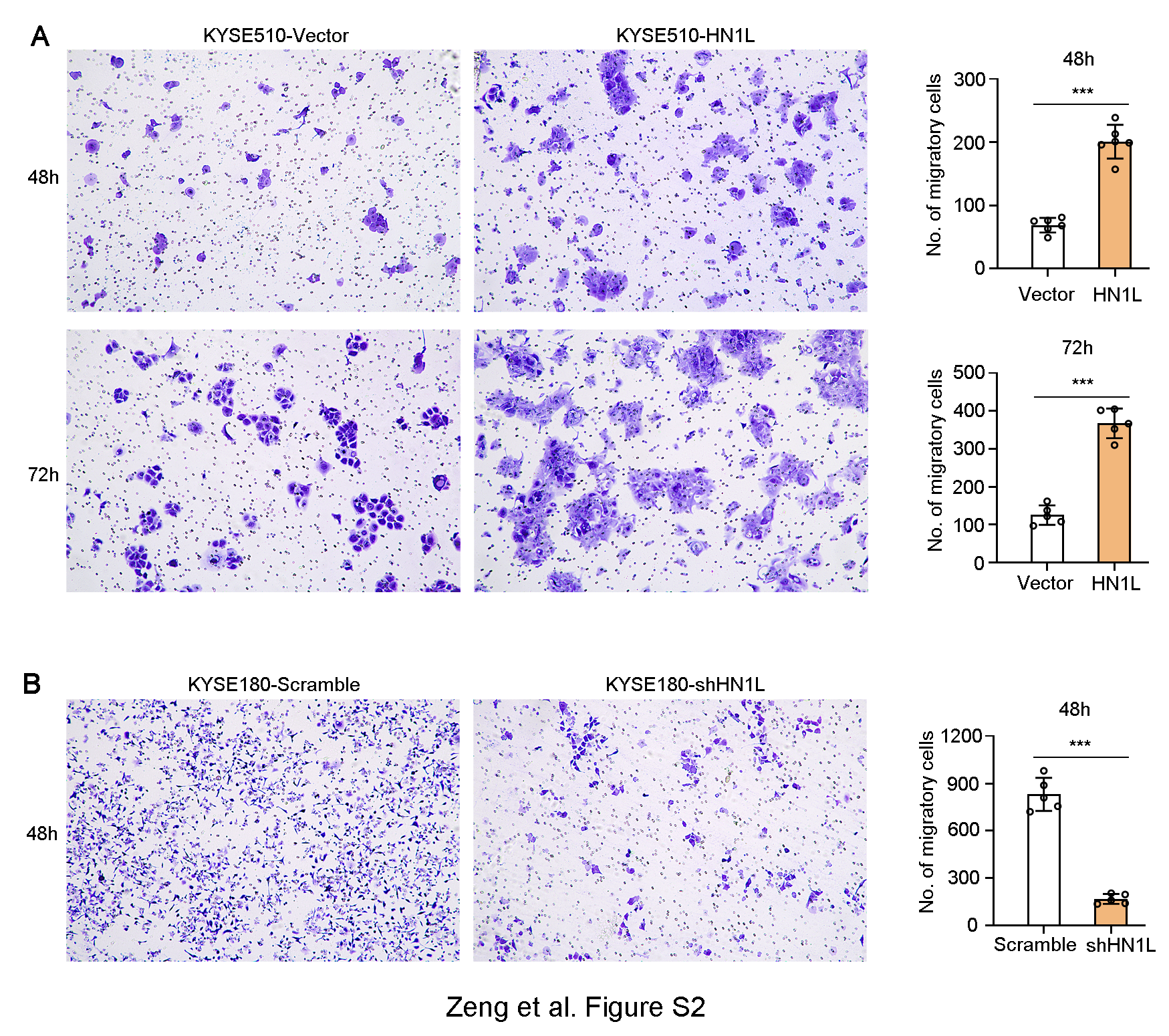

Supplement: Supplementary file 3 — Supplementary Figure S2 [file 41419_2022_5478_MOESM3_ESM.tif]

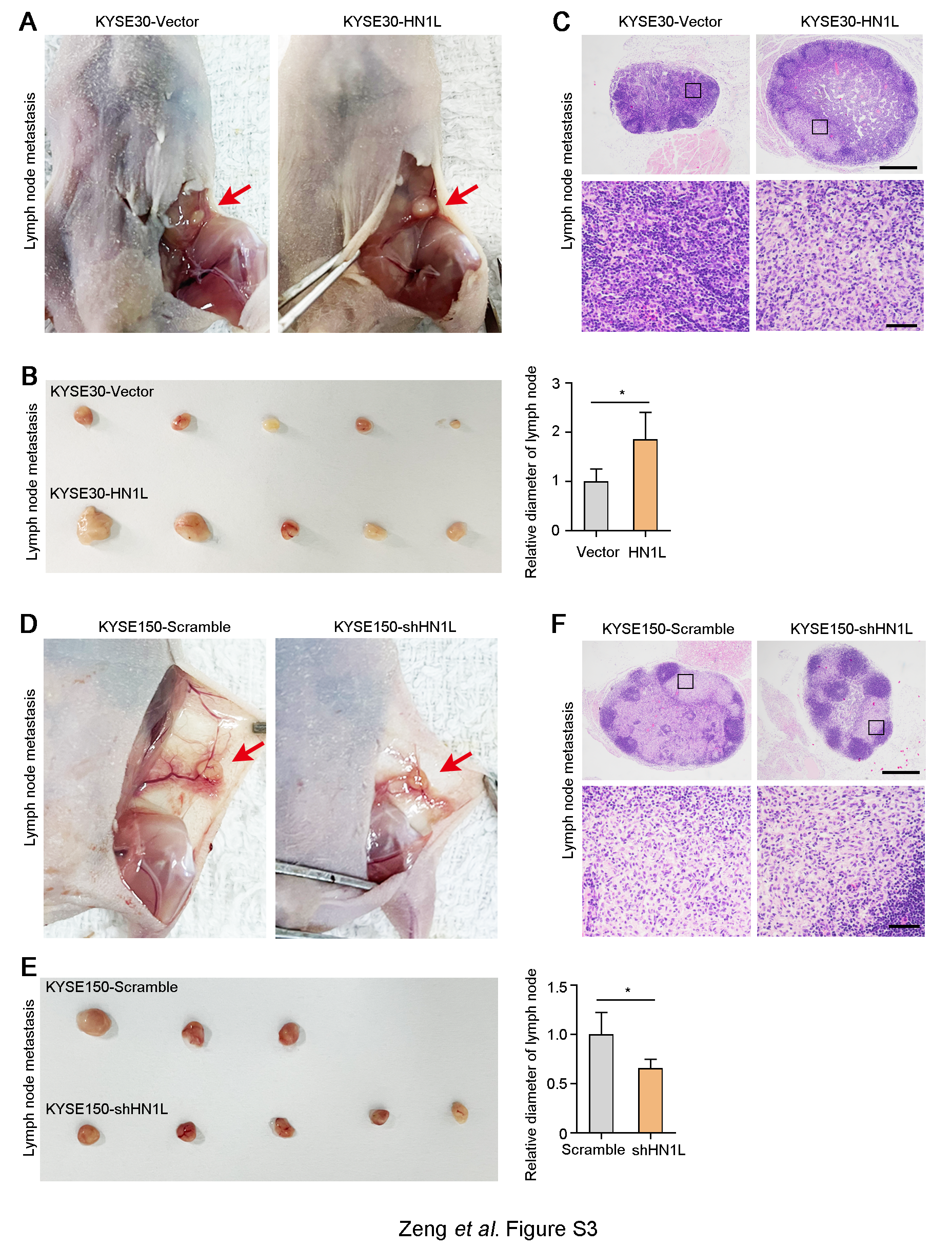

Supplement: Supplementary file 4 — Supplementary Figure S3 [file 41419_2022_5478_MOESM4_ESM.tif]

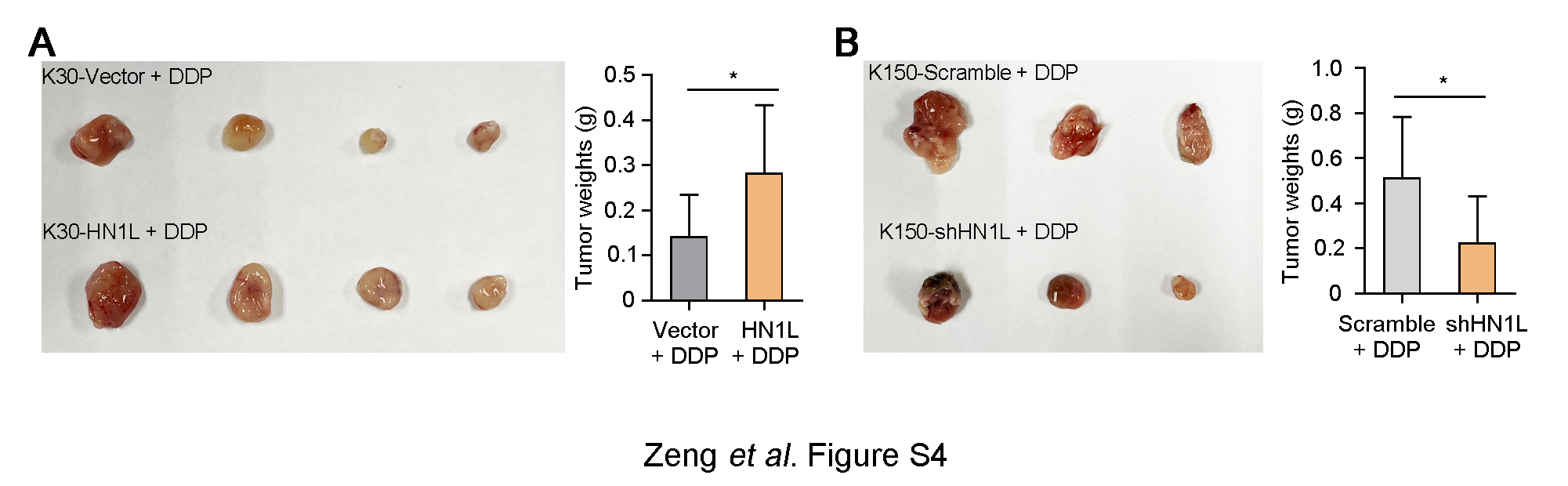

Supplement: Supplementary file 5 — Supplementary Figure S4 [file 41419_2022_5478_MOESM5_ESM.tif]

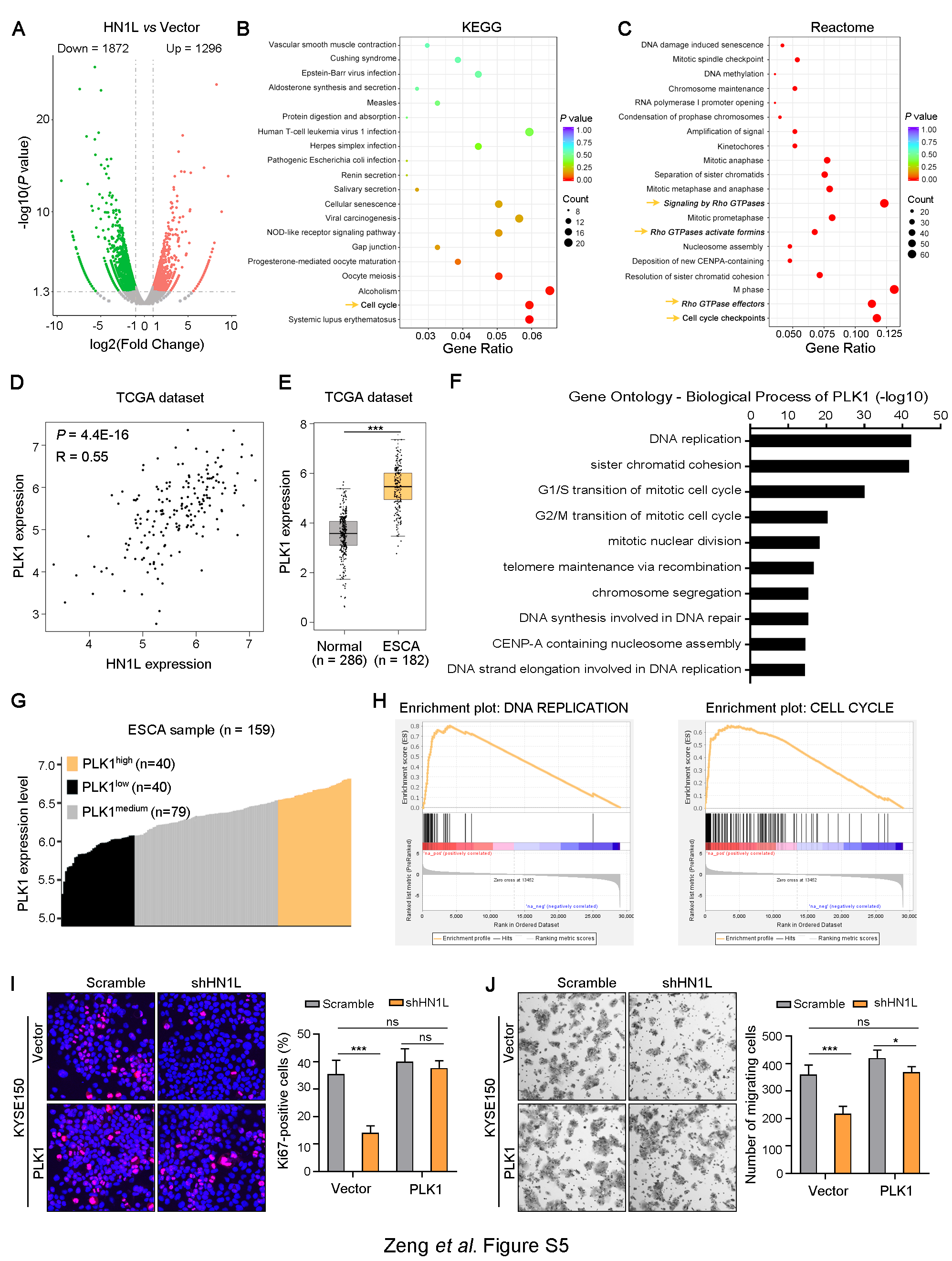

Supplement: Supplementary file 6 — Supplementary Figure S5 [file 41419_2022_5478_MOESM6_ESM.tif]

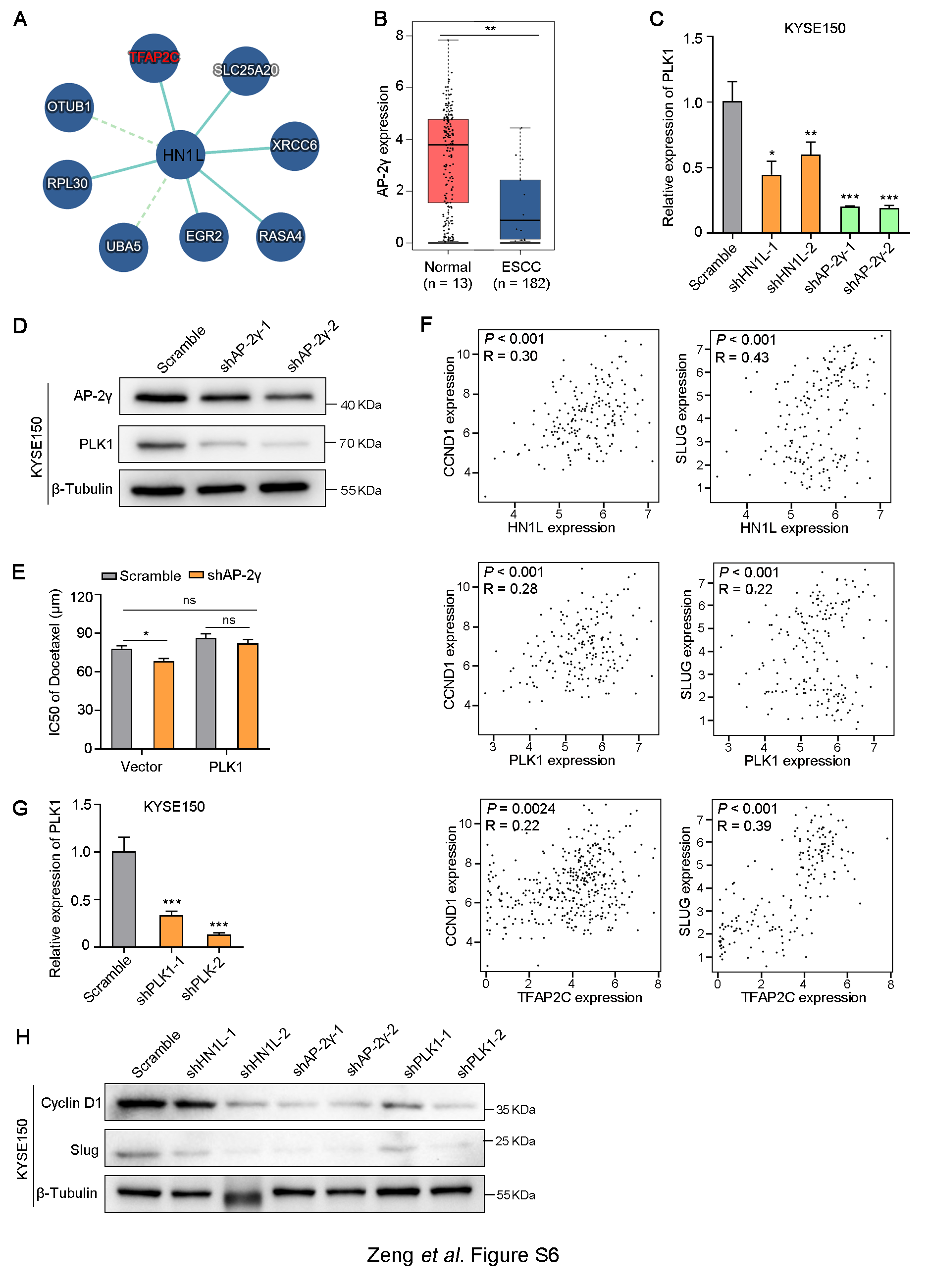

Supplement: Supplementary file 7 — Supplementary Figure S6 [file 41419_2022_5478_MOESM7_ESM.tif]
