# Supplementary material for: HN1L/AP-2γ/PLK1 signaling drives tumor progression and chemotherapy resistance in esophageal squamous cell carcinoma
Source: Cell Death Dis. 2022 Dec 7;13(12):1026. doi: 10.1038/s41419-022-05478-1 (PMC9729194; doi:10.1038/s41419-022-05478-1)
Supplement: Supplementary file 8 — Supplementary Table S1 [file 41419_2022_5478_MOESM8_ESM.docx]

**Table S1. Nucleotide sequence used for gene knockdown or qPCR**

| **shRNA** | **Sequence (5'-3')** |
| --- | --- |
| Scramble | GCTTCGCGCCGTAGTCTTA |
| shHN1L-1 | GGCGTAAGCAGAAACACTAAC |
| shHN1L-2 | GGTGGAAATGAACTACCATTT |
| shAP-2γ-1 | GCCGAATTTCCTAGTAAACCA |
| shAP-2γ-2 | GGGAAGAGTTTGTTACCTACC |
| shPLK1-1 | GGTATCAGCTCTGTGATAACA |
| shPLK1-2 | CCTTGATGAAGAAGATCACCC |
| **Primer** | **Sequence (5'-3')** |
| PLK1 | AAGTACGGCCTTGGGTATCA (forward) |
|  | TCAGTGGGCACAAGATGAGC (reverse) |
| PLK1 promoter | CTGACCAAGAAACTGAGTGT(forward) |
|  | CGAAGCTGTCTGGCCTCCTC (reverse) |
