# Supplementary material for: HN1L/AP-2γ/PLK1 signaling drives tumor progression and chemotherapy resistance in esophageal squamous cell carcinoma
Source: Cell Death Dis. 2022 Dec 7;13(12):1026. doi: 10.1038/s41419-022-05478-1 (PMC9729194; doi:10.1038/s41419-022-05478-1)
Supplement: Supplementary file 9 — Supplementary Table S2 [file 41419_2022_5478_MOESM9_ESM.docx]

| **Antibodies** | **Corporations** | **Catalog** | **Dilutions** |
| --- | --- | --- | --- |
| HN1L | Abcam | #ab200571 | 1:4,000 |
| PLK1 | Cell Signaling Technology | # 4513 | 1:1,000 |
| Cyclin D1 | Cell Signaling Technology | #2922 | 1:1,000 |
| Slug | Cell Signaling Technology | # 9585 | 1:1,000 |
| GAPDH | Cell Signaling Technology | #5174 | 1:3,000 |
| β-Tubulin | Cell Signaling Technology | #2128 | 1:2,000 |

**Table S2. Primary antibodies that used in western blot analysis**
