# Supplementary material for: HN1L/AP-2γ/PLK1 signaling drives tumor progression and chemotherapy resistance in esophageal squamous cell carcinoma
Source: Cell Death Dis. 2022 Dec 7;13(12):1026. doi: 10.1038/s41419-022-05478-1 (PMC9729194; doi:10.1038/s41419-022-05478-1)
Supplement: Supplementary file 10 — Supplementary Table S3 [file 41419_2022_5478_MOESM10_ESM.docx]

| **Uniprot ID** | **Protein** | **Curation Events** |
| --- | --- | --- |
| P12956 | XRCC6 | 3 [IntAct/HPRD/StelzlLow] |
| O43374 | RASA4 | 3 [IntAct/HPRD/StelzlLow] |
| O43772 | SLC25A20/CACT/CAC | 3 [IntAct/HPRD/StelzlLow] |
| Q9GZZ9 | UBA5 | 2 [IntAct/BioGRID] |
| P11161 | EGR2 | 2 [IntAct/BioGRID] |
| Q86X55 | CARM1 | 2 [BioGRID/BioPlex] |
| Q6PIW4 | FIGNL1 | 2 [BioGRID/BioPlex] |
| P48730 | CKID | 2 [BioGRID/MINT] |
| Q15019 | SEPT2 | 2 [BioGRID/MINT] |
| P26992 | CNTFR | 2 [BioGRID/MINT] |
| Q92754 | **TFAP2C** | 1 [IntAct] |
| Q96FW1 | OTUB1 | 1 [IntAct] |
| P02545 | LMNA | 1 [BioGRID] |
| Q9NZC7 | WWOX | 1 [BioGRID] |
| P04792 | HSPB1 | 1 [BioGRID] |
| P16949 | STMN1 | 1 [BioGRID] |

**Table S3. Proteins interacting with HN1L**
