# Supplementary material for: HN1L/AP-2γ/PLK1 signaling drives tumor progression and chemotherapy resistance in esophageal squamous cell carcinoma
Source: Cell Death Dis. 2022 Dec 7;13(12):1026. doi: 10.1038/s41419-022-05478-1 (PMC9729194; doi:10.1038/s41419-022-05478-1)

Figure 1

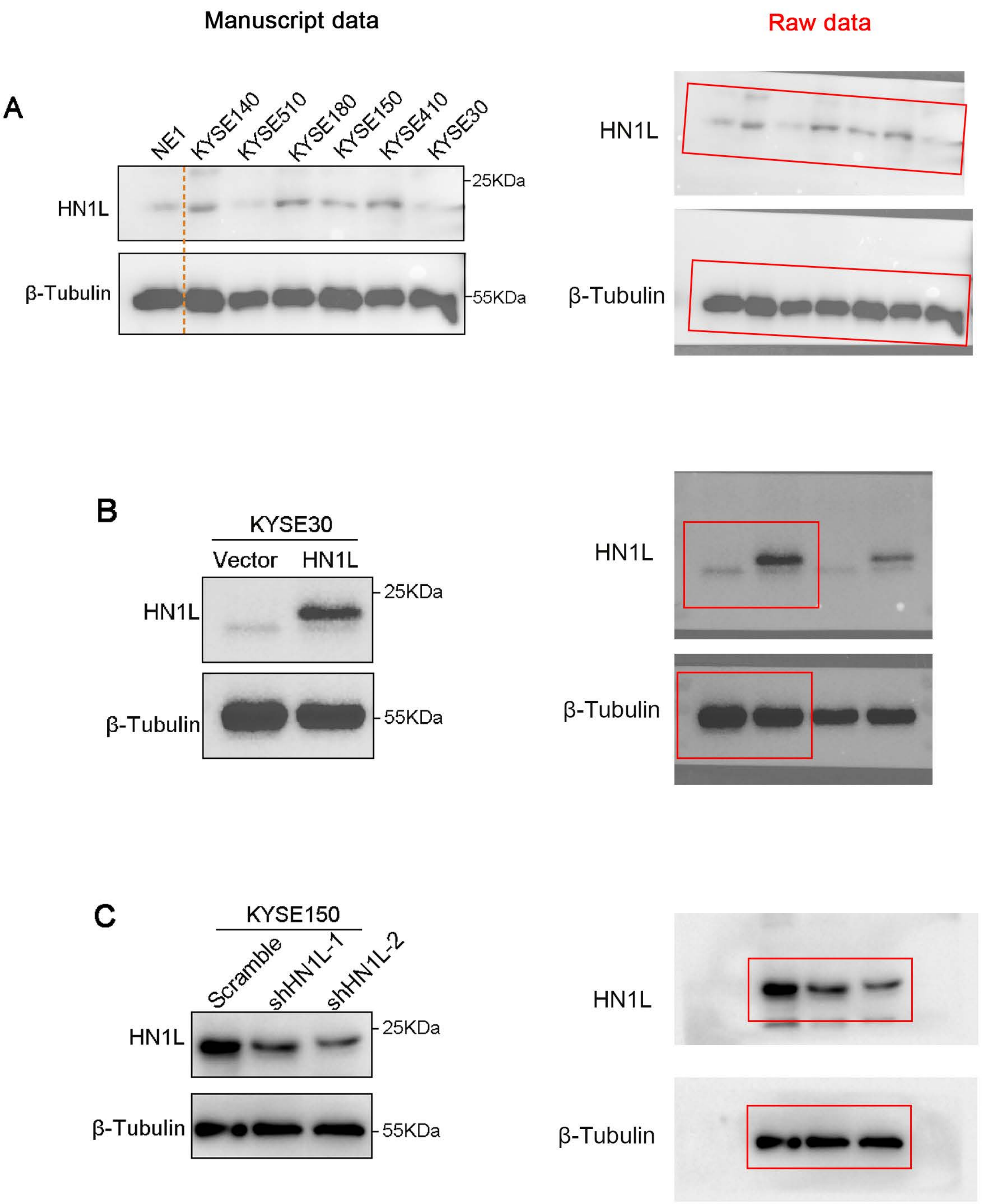
Manuscript data
Raw data
A
NE1
KYSE140
KYSE510
KYSE180
KYSE150
KYSE410
KYSE30
HN1L
β-Tubulin
25KDa
55KDa
HN1L
β-Tubulin
B
KYSE30
Vector
HN1L
HN1L
β-Tubulin
25KDa
55KDa
HN1L
β-Tubulin
C
KYSE150
Scramble
shHN1L-1
shHN1L-2
HN1L
β-Tubulin
25KDa
55KDa
HN1L
β-Tubulin

Figure 5

Manuscript data

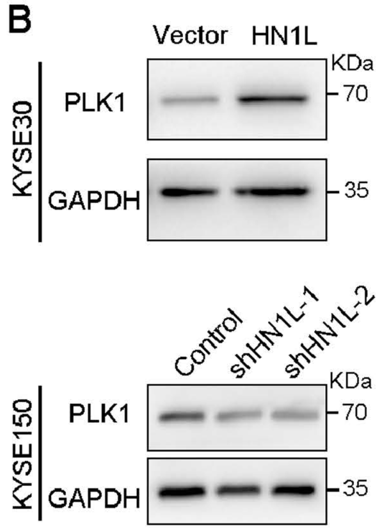

Raw data

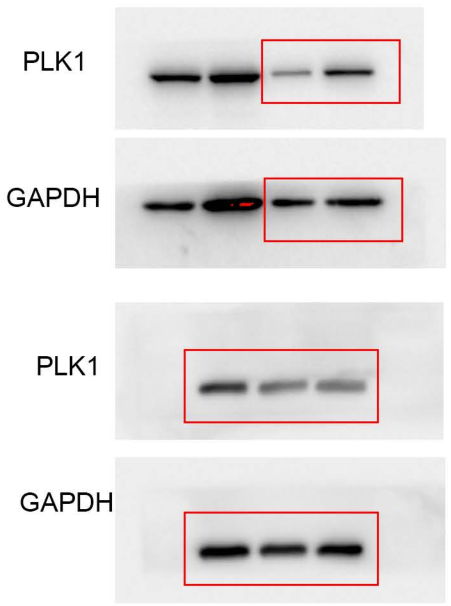

Figure 6

Manuscript data

Raw data

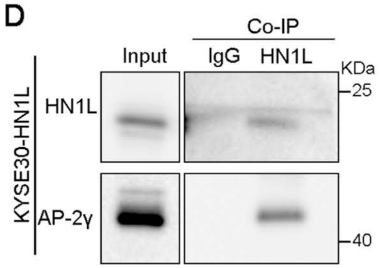

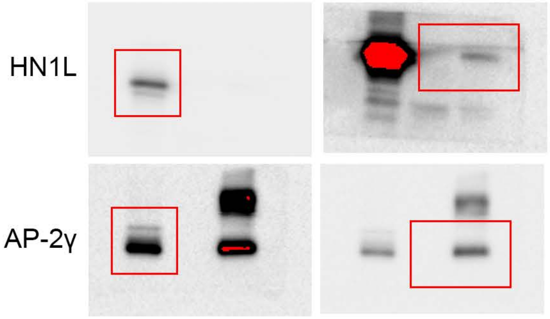

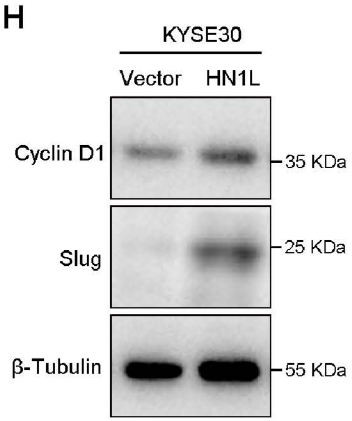

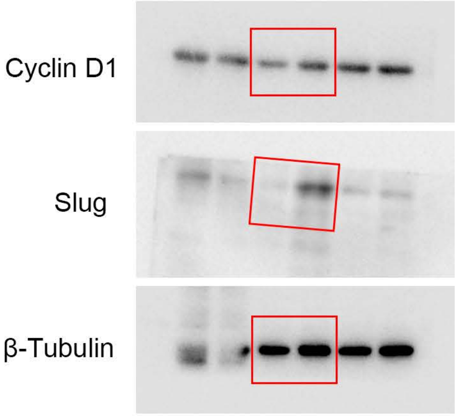

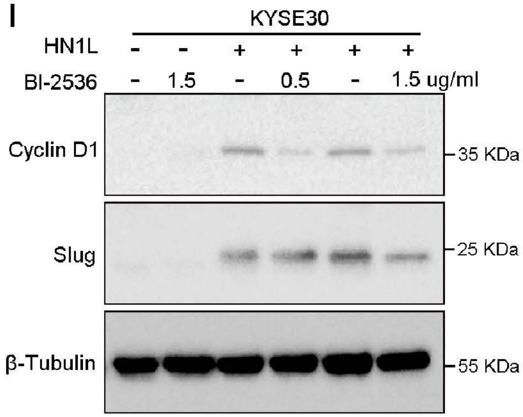

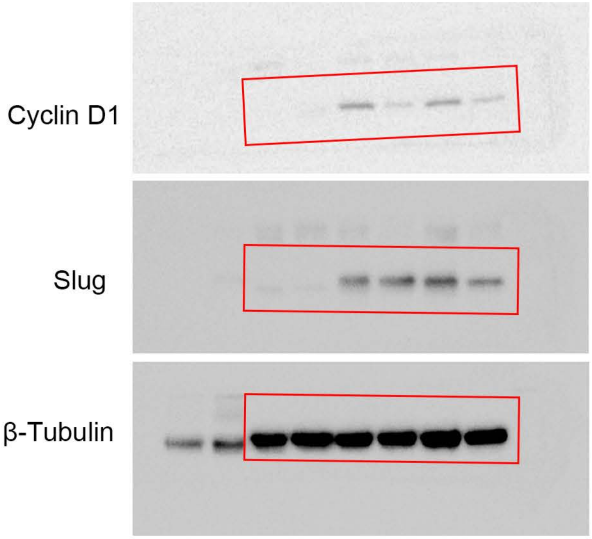

Supplement: Supplementary file 12 — Raw data of western blots [file 41419_2022_5478_MOESM12_ESM.pdf]
